# Comprehensive geriatric assessment in elderly outpatients with dementia

**DOI:** 10.1590/S1980-57642008DN10300013

**Published:** 2007

**Authors:** Márlon Juliano Romero Aliberti, Elina Lika Kikuchi, Regina Miksian Magaldi, Sérgio Márcio Pacheco Paschoal, Wilson Jacob Filho

**Affiliations:** 1 Adjunct Physician of Geriatric Day Hospital. Geriatric Division of Hospital das Clínicas of Department of Internal Medicine of São Paulo University School of Medicine São Paulo, SP, Brazil.; 2 Adjunct Physician of Geriatric Service Geriatric Division of Hospital das Clínicas of Department of Internal Medicine of São Paulo University School of Medicine São Paulo, SP, Brazil.; 3 Professor of Geriatric Service Geriatric Division of Hospital das Clínicas of Department of Internal Medicine of São Paulo University School of Medicine São Paulo, SP, Brazil.

**Keywords:** elderly, dementia, geriatric assessment

## Abstract

Dementia is a common disabling disease in the elderly. In such patients, general health conditions may worsen the functional decline and loss of autonomy. The Comprehensive Geriatric Assessment (CGA) is a validated and recommended instrument for multidimensional evaluation of the aged. Nonetheless, it has yet to be assessed in demented patients.

**Objectives:**

To analyze the functional, emotional and clinical status in elderly with dementia measured by the CGA. We also compared the results obtained in the same patients stratified for severity of dementia.

**Methods:**

Transversal study with demented elderly outpatients. Subjects were evaluated by the CGA consisting of clinical data, Clinical Dementia Rating, performance-oriented mobility assessment of gait and balance (POMA), Cornell scale for depression, activities of daily living, Mini Mental Status Examination (MMSE), Mini Nutritional Assessment, Whispered and Snellen Test.

**Results:**

Fifty-two patients with mean age of 77 years were evaluated. Majority of patients had Alzheimer disease (77%). Depression was the most prevalent comorbidity. The POMA score was related to the number of falls in the previous year. Also, there was correlation between complaints of visual and hearing impairment and the results on the Snellen and Whispered Tests. Regarding severity, 56% presented mild, 33% moderate and 11% severe condition. Patients with moderate/severe dementia had less leisure activities, greater risk of falls, along with worse performance on the MMSE, POMA and activities of daily living.

**Conclusions:**

The CGA was applied in demented elderly with the help of their caregivers, and was able to better characterize patient state of health. Subjects with moderate/severe dementia obtained poor results in several assessed criteria.

## Abstract (pt)

Demência é uma doença comum e incapacitante entre os idosos. Nesses pacientes, condições gerais de saúde podem agravar ainda mais o declínio funcional e a perda da autonomia. A Avaliação Geriátrica Global (AGG) é um instrumento validado e recomendado para uma abordagem multidimensional do idoso, porém, ainda pouco estudada em pacientes com demência.

**Objetivo:**

Analisar o estado funcional, emocional e clínico dos idosos portadores de demência através da aplicação da AGG. Nós também comparamos os resultados obtidos nos mesmos pacientes classificados de acordo com a gravidade da demência.

**Métodos:**

Estudo transversal com idosos ambulatoriais portadores de demência. Eles, acompanhados de seus cuidadores, foram submetidos à AGG que constava de dados clínicos, escore clínico de demência, escala do equilíbrio e marcha de Tinetti (EEMT), escala de Cornell para depressão, atividades de vida diária, mini exame do estado mental (MEEM), mini avaliação nutricional e Testes do Sussurro e Snellen.

**Resultados:**

Cinqüenta e dois idosos com idade média de 77 anos. A maioria possuía doença de Alzheimer (77%). A comorbidade mais prevalente foi depressão. A pontuação na EEMT relacionou-se com o número de quedas no último ano. Houve também correlação entre a queixa de déficit auditivo e visual com os resultados dos Testes do Sussurro e Snellen. Em relação à gravidade obteve-se: 56% leve, 33% moderada e 11% grave. Os pacientes com demência moderada/grave apresentaram menos atividades de lazer, maior risco para quedas, pior desempenho no MEEM, na EEMT e nas atividades de vida diária.

**Conclusões:**

A AGG foi realizada nos idosos portadores de distúrbios cognitivos com o auxílio do cuidador, e foi capaz de caracterizar melhor o estado geral de saúde desses pacientes. Indivíduos com demência moderada/grave obtiveram piores resultados em diversos critérios avaliados.

Regaining or maintaining a good quality of life is one of the main objectives of geriatric clinical management.^1^ Currently, the aging process is associated with increased prevalence of multiple diseases and disabilities. It is estimated that up to 50% of very old subjects, over 85 years of age, are dependent in daily activities.^2^

Dementia is a common disease among the elderly, and is related to functional progressive decline and gradual loss of autonomy from its early stages. In the United States, for instance, dementia is the third major cause of disability and mortality and has severe consequences for health.^3-6^

While there have been improvements in scientific knowledge concerning diagnosis, treatment and management of behavioral and cognitive symptoms of patients with dementia, medical literature does not provide enough data on their general health condition. Demented patients also present common elderly health problems (multiple chronic diseases, depression and geriatric syndromes) that may worsen loss of independence and autonomy. Recent studies suggest that this group presents more comorbidities^4-7^ and less survival, associated with the presence of sensorial impairment, gait disorders, falls, heart failure, and *diabetes mellitus*.^8^

Identifying clinical alterations and disabilities among elderly patients is not straightforward. The task becomes even more complex if the patient has cognitive disorders, mainly because of the difficulty in reporting symptoms and adverse effects.

The Comprehensive Geriatric Assessment (CGA) is an instrument used in Geriatrics that is able to play such a role. Furthermore, it is a multidimensional process, which covers social, emotional, cognitive and physical parameters, emphasizing quality of life and functional ability. Also, the CGA provides a general aspect of the aged and contributes to treatments and long-term follow-ups.^9-12^

Following the emergence of Geriatrics, the CGA started being used in the United Kingdom in the 1930s by Marjory Warren. Since then, this assessment has been widely studied and improved.^12^

Randomized studies with elderly have shown better diagnostic accuracy and survival, reduced health expenses as well as medical care in emergency departments. Also, there was less risk of admission to a nursing home.^9^ In-patient comprehensive geriatric assessment (CGA) may reduce short-term mortality, increase the chances of living at home at one year, and improve physical and cognitive function.^11^

This assessment may be applied differently according to the health team and location where it is administered. Frequently, it is complemented by several quantitative, validated, easily applicable scales that assess geriatric syndromes.^9,10^

This evaluation involves the patient’s decision-making capacity, level of understanding information, and ability to communicate one’s choices. In the elderly with dementia, such capabilities may be preserved during the initial stage. Nevertheless, with the progression of the disease, these often become obstacles.^3,4^ For this reason, scant data is available about CGA in demented patients. Several studies consider dementia as an exclusion criterion. Therefore, not only in these cases, the support of relatives and caregivers could be useful to obtain correct information.^9^

In the context of elderly with dementia, all the variables that could be related to loss of autonomy must be examined. We believe that frail elderly, such as demented individuals, should be screened through the standardized tools of the CGA to aid diagnosis, assessment and, recommendably, rehabilitation and outcome measurements.

This study proposed to analyze the functional, emotional and clinical status in elderly outpatients with dementia evaluated by the tools of the CGA. We also compared the results obtained by the CGA in the same patients stratified for mild, moderate and severe dementia. Our secondary aim was to underline that multidimensional assessment is effective to detect underestimated clinical difficulties in demented patients.

## Methods

The study was carried out with outpatients from the Center of Cognitive Disorders (CEREDIC) of Hospital das Clínicas of São Paulo University School of Medicine (HCFMUSP) between October 1^st^ and November 30^th^ 2005. The inclusion criteria were: age ≥60 years, diagnosis of dementia, and current follow-up by CEREDIC. Dementia diagnosis was made in accordance with the criteria of the Diagnostic and Statistical Manual of Mental Disorders – Fourth Edition (DSM-IV).

The project was approved by the Commission of Ethics of HCFMUSP and an informed consent was signed by all patients or their caregivers before their inclusion in the study.

This was a transversal study with a convenient sample initially reexamining 110 medical records in an ascending numerical order, which constituted part of the CEREDIC archive. In this process, fifty medical records were not included because the patients had non-dementia causes underlying their cognitive decline (59%), were not in current follow up (39%), or were younger than sixty years (2%).

**Figure 1:**
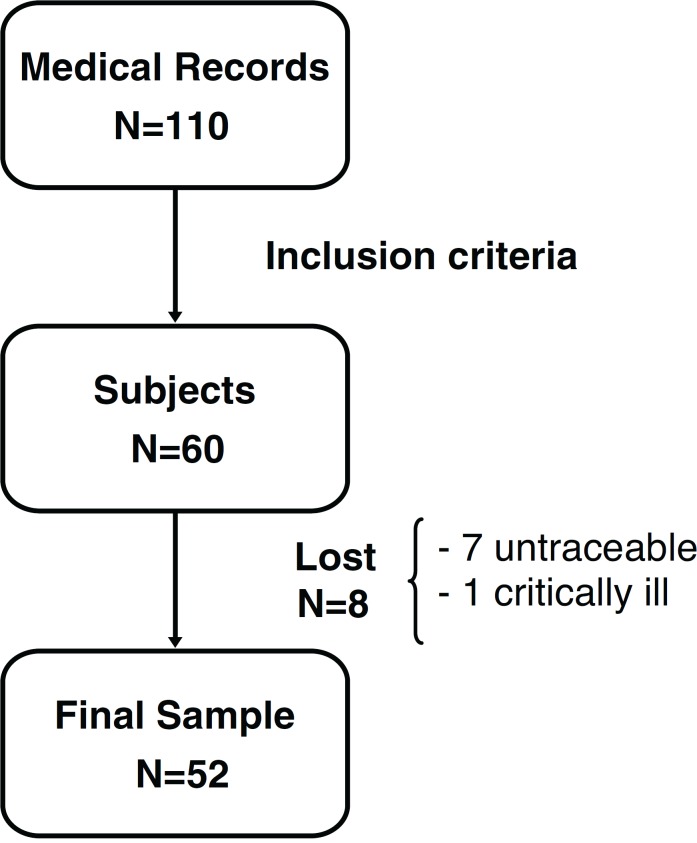
Sample construction.

In addition, these patients were invited to be evaluated at CEREDIC, by a telephone call to a relative or caregiver. The commitment to take part occurred only after verbal consent of the subjects or their caregivers. We were not able to contact seven patients by phone and one was critically ill. Figure 1 shows the process of building the sample.

**Figure 2:**
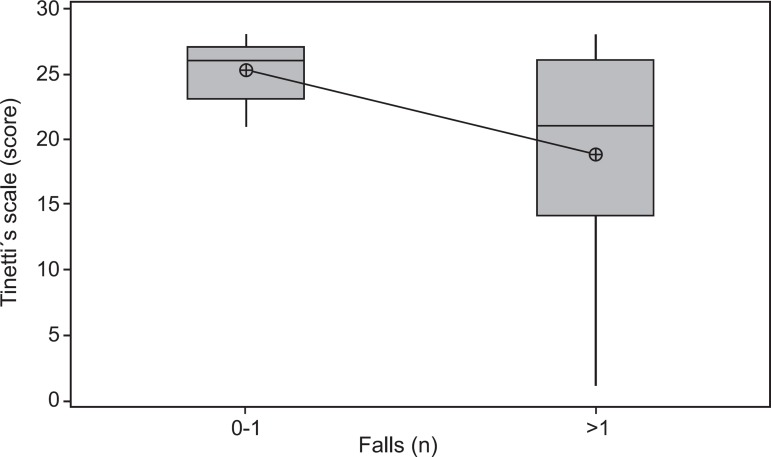
Correlation between score on Tinetti´s scale and number of falls in the last year.

On the day of the assessment, the patients – together with their caregivers– were submitted to a research tool called the “Comprehensive Geriatric Assessment” (Figure 2), which lasted approximately 45 minutes. The CGA was performed by a trained geriatrician.

The CGA consisted of identification (personal data), reporting of comorbidities, list of medication used, vaccination status, level of physical activity and leisure, Clinical Dementia Rating (CDR) scale,^13^ scale of performance-oriented mobility assessment of gait and balance (POMA) of Tinetti,^14^ Cornell scale for depression,^15^ Katz scale of basic activities of daily living (BADL),^16^ Lawton’s scale of instrumental activities of daily living (IADL),^17^ Mini Mental Status Examination (MMSE),^18^ Mini Nutritional Assessment (MNA),^19^ Whispered Test and Snellen Test.

This instrument was devised based on the CGA developed by the Brazilian Society of Geriatrics and Gerontology and approved by the Brazilian Medical Association in September of 2005, as a medical procedure, during the fourth edition of *Classificação Hierarquizada de Procedimentos Médicos*.^20^ The modifications were made in order to adapt the tool for the study of patients with cognitive impairment.

Caregivers helped by giving a range of data, such as: comorbidities, medication used, vaccination status, level of physical activity and leisure, number of falls in the previous year, and presence of sensorial complaints. The Cornell scale for depression, Katz and Lawton scales, along with the MNA were also administered with their help.

### Statistical analysis

The MINITAB 14 statistical program was used for statistical analysis. Descriptive statistics were calculated for quantitative variables. Furthermore, tests of equality of means were performed. Double-entry tables were presented and chi-square tests were applied in order to compare distributions for qualitative variables. In addition, the sample was divided into two groups (called A and B). When the suppositions of this test were not fulfilled, the verisimilitude ratio tests were applied or, for the case of two-by-two tables, Fisher’s Exact Tests. In this study, p=0.05 was considered statistically significant.

## Results

A total of 52 aged were assessed in this study, 35 (67.3%) women and 17 (32.7%) men, with mean age of 76.9 (±6.0) years and 5.3 (±4.5) years of schooling.

Regarding marital status, 23 patients (44.2%) were married, 22 (42.3%) were widowed, 4 (7.7%) were single and 3 (5.8%) were divorced. Among the widowed subjects, almost all (95.5%) were women. Forty (76.9%) elderly patients were retired.

The main caregivers identified were mostly the children, responsible for 27 (52.9%) patients, followed by spouses, responsible for 16 (31.3%). Other caregivers were siblings, a grandchild, neighbor, and professionals. Only one patient did not have a caregiver.

The main cause of dementia was Alzheimer disease in 40 (76.9%) cases, followed by 7 (13.5%) cases of mixed dementia and 2 (3.9%) of vascular causes. Fronto-temporal, Lewy body and potentially reversible dementias each presented in 1 (1.9%) case.

The mean MMSE was 17.4 (±6.8) ranged from 0 to 30. The severity of the disease was evaluated by the CDR scale. According to this analysis, 29 (55.8%) patients had mild dementia (CDR 1), 17 (32.7%) moderate (CDR 2) and 6 (11.5%) severe dementia (CDR 3).

The mean number of comorbidities was 3.0 (±1.4), with a range from 0 to 6. The main comorbidity detected was depression, present in half of the aged patients. The mean number of drugs administered was 5.0 (±2.2) with a range from 1 to 12. There was a high prevalence (71.1%) of polypharmacy, defined as the use of four or more drugs.^21^

Regarding vaccination status, the characteristics of the sample were: 38 (73.0%) subjects were properly vaccinated against influenza, 22 (42.3%) against pneumococcus, and 28 (53.8%) against tetanus.

The only leisure activity of 9 (17.3%) patients was watching TV. Only 14 (26.9%) of the sample practiced any kind of physical activity. In addition, majority of these (92.8%) tended to go jogging.

Scores from the POMA analysis revealed that 15 (28.8%) patients had moderate risk of falls (19-24 points) and 7 (13.5%) high risk (<19 points). Moreover, 6 (11.7%) patients reported the use of some assistive devices to walk. There was statistical correlation between the number of falls in the previous year and the performance on this scale (p=0.007) (Figure 2).

A number of patients reported hearing (46.1%) and visual (69.2%) impairment. An altered Whispered Test was detected in 45.1% of the subjects, and 70.8% presented an altered Snellen Test. The subjective complaints of hearing and visual impairment correlated with results on the Whispered Test (p<0.001) and Snellen test (p=0.002), respectively. The performing of the Whispered Test was not applicable in one patient with severe dementia. Moreover, the Snellen test could not be applied in four patients – one of these had moderate dementia and three had severe dementia.

The Cornell scale demonstrated symptoms of depression in 24 (46%) patients: half of these having mild depression (score ≥8 points) and the other half with moderate depression (score ≥12 points).

The functional assessment was determined by basic activities (Katz) and instrumental activities (Lawton) on daily living scales (Table 1).

**Table 1:** Performance of the elderly patients according to scales of activities of daily living.

	Katz scale	Lawton scale
Level of dependence*	(BADL)	(IADL)
Dependent (D)	10 (19.2%)	26 (50.0%)
Partially dependent (P)	17 (32.7%)	24 (46.1%)
Independent (I)	25 (48.1%)	2 (3.9%)

* Score on Katz Scale (BADLs): D (0 to 3 points), P (4 or 5 points), and I (6 points); * Score on Lawton Scale (IADLs): D (9 to 15 points), P (16 to 25 points), and I (26 and 27 points)

Nutritional status was assessed using the score of MNA. This study showed 38 (73.0%) elderly patients to be nourished (≥12 points on short form or ≥24 points on total score), 12 (23.0%) at risk of malnutrition (17-23.5 points on total score) and only 2 (4.0%) malnourished subjects (<17 points on total score). The two malnourished patients presented severe dementia.

The sample was divided into two groups to verify differences in performance on the CGA in relation to the severity of the dementia: Group A (patients with CDR 1) and Group B (patients with CDR 2 and 3). The reduced number of subjects with severe dementia (11.5%) led to this analysis in only two groups.

Tables 2 and 3 show general characteristics and compare both groups.

**Table 2:** General characteristics of groups A and B*.

Variables	Group A (n=29)	Group B (n=23)	p
Age (years)	78.0 (±5.1)	75.6 (±6.9)	0.182
Schooling Level (years)	5.0 (±3.8)	5.7 (±5.4)	0.619
Comorbidities (number)	3.0 (±1.4)	2.9 (±1.5)	0.772
Medications (number)	4.6 (±1.8)	5.5 (±2.6)	0.173
MMSE (score)	21.5 (±3.8)	12.3 (±6.4)	<0.001^†^
Leisure Activities (number)	3.4 (±1.6)	2.4 (±1.5)	0.028^†^
Falls (number in the previous year)	0.6 (±1.2)	3.0 (±6.3)	0.082
Tinetti Scale (score)	25.1 (±3.1)	21.2 (±6.8)	0.018^†^

* Data presented for mean and standard deviation of each group;

† Values of p≤0.05.

**Table 3:** Comparative variables between groups A and B.

Variables	Group A (n=29)	Group B (n=23)	p
Dementia - Alzheimer's type	25 (86.2%)	15 (65.2%)	0.074
Adequate vaccination status			
Influenza	21 (72.4%)	17 (73.9%)	0.541
Pneumococcus	13 (44.8%)	9 (39.1%)	0.471
Tetanus	16 (55.2%)	12 (52.2%)	0.507
Practice of regular physical activities	9 (31.0%)	5 (21.7%)	0,538
Moderate risk of falls (Tinetti scale)	8 (2.7%)	7 (30.4%)	0.822
High risk of falls (Tinetti scale)	1 (3.4%)	6 (26.0%)	0.018*
Dependent for BADLs^†^	0 (0.0%)	10 (43.4%)	<0.00*
Dependent for IADLs^‡^	7 (24.1%)	19 (82.6%)	<0.001*
Depression (Cornell scale)	10 (34.4%)	14 (60.8%)	0.058
Risk of malnutrition (MNA)^§^	6 (20.6%)	6 (28.5%)^¶^	0.520
Altered whispered test	12 (41.3%)	11 (50.0%)^#^	0.540
Altered Snellen test	21 (72.4%)	13 (68.4%)**	0.766

* p≤0.05;

† Basic activities of daily living;

‡ Instrumental activities of daily living;

§ Mini nutritional assessment;

¶ considering n=21;

# considering n=22;

** considering n=19.

These two tables indicate that the elderly patients in Group B had a worse performance compared to subjects in Group A regarding some variables. There were a lower number of leisure activities, higher risk of falls, and higher rates of dependency, characterizing greater functional impairment. This group also obtained lower scores on the MMSE, reflecting the most severe cognitive impairment of these patients. Also, the subjects in Group B tended to present symptoms of depression (p=0.056), according to values obtained on the Cornell scale.

## Discussion

Elderly with dementia are often considered exclusion factors for several studies involving the CGA because cognitive alterations make this assessment more complex and difficult.^9^ Nonetheless, this study confirmed that the CGA can be applied to elderly outpatients with dementia. Furthermore, the tool used showed different aspects of their health.

It is important to emphasize that the assistance of the caregiver was essential in completing the whole process. The initial contact and invitation by telephone call depended on them. Moreover, they took the patients to CEREDIC and also participated actively in the interview being the source of several items of data.

As reported earlier, the subjects with dementia become dependent on others, normally relatives.^3^ In the sample studied, only one elderly patient, with mild dementia, did not have a caregiver. Moreover, 84.2% of the caregivers were their children or spouse. Family creates a unique entity with the patient and provides information about the patient. Besides, the relatives participate and help monitoring all interventions of the health team.^22^ Therefore, it is important to dedicate attention to relatives, who also suffer the consequences of the disease.

Our population was composed largely of women with Alzheimer disease (AD). Such data correlates with those in the medical literature, which show prevalence of this disease among female subjects (approximate proportion of 2:1)^3^ and that AD is responsible for 50 to 60% of the cases of dementia among the aged.^23^

Other important characteristics of the sample were: low schooling level and high number of comorbidities. The group reflects the section of the Brazilian general elderly population that was unable to study when young. In addition, these individuals are part of the current aging process, which is associated with the presence of several chronic diseases.^24^

Elderly patients with cognitive disorders tend to develop adverse drug reactions (ADR). In this study, a high prevalence of polypharmacy (71.1%) was observed, which represents an important risk factor for developing ADR. Prescribing only the necessary drugs, at adequate dosage, and suggesting the supervision of the caregiver regarding the use of medicines, are procedures that can minimize the effects of polypharmacy on these patients.^21,25^

An important cause of mortality in the elderly with dementia is infection, pneumonia being the main example.^26^ Vaccination against influenza and pneumococcus reduces risks of respiratory infections among the aged. Besides, it reduces the severity of these cases, the number of hospitalizations, and mortality.^27^ There are no literature data specifically about patients with dementia. This study showed that 73% of the elderly patients had been properly vaccinated against influenza and only 42.3% against pneumococcus, independent of their severity. In 2004, 85% of the Brazilian elderly population was vaccinated against influenza.^28^ As they are fragile patients with regular medical follow-up, a wider vaccination coverage was expected. Furthermore, individuals not properly vaccinated must be made aware of the possible risks of this situation and must be referred for vaccination.

Leisure and physical activity are elements considered by the CGA, whereas these are usually ignored by traditional assessments. Although such activities were barely practiced by the elderly patients evaluated, especially those with moderate and severe disease, such activity may provide patients with well-being, improvement in health, and efficient social interaction.

Dementia is an independent risk factor for falls.^29^ In this study, an elevated rate of 42.3% of risk of falls was observed among the elderly. Several characteristics and deficiencies may be related to this increase in risk of falls: spatial disorientation, adaptation difficulties to new environments because of attention and memory impairment, gait impairment and postural instability, poor judgment of this clinical feature and low capacity to acknowledge and avoid hazards. These factors worsen with the severity of the dementia, increasing the risk of falls with progression of the disease. Measures such as assistive devices, physical activity, physical therapy, adjustment of the environment, and treatment of osteoporosis are very efficient, diminishing the impact of falls and their consequences.

Sensorial impairments, often easily correctable, are not given weight and may imply great functional and cognitive impairments. These subjects reported a high percentage of visual (69.2%) and hearing (46.1%) complaints, which were confirmed by easily applied screening tests (Whispered test and Snellen test, respectively), except in some moderate and severe demented patients. Referral to a specialist for a detailed assessment and treatment can yield benefits for these cases.

Among comorbidities, depression is an important clinical condition that leads to poor evolution, high number of hospitalizations, and high mortality of patients. The prevalence of depressive disorders in patients with dementia varies from 0 to 86%, with an average of 19%. The diagnosis is difficult because its symptoms are often part of the clinical features.^30,31^ In this study, depression was the most prevalent comorbidity. In addition, according to data obtained using the Cornell scale, half of the patients with previous diagnosis of depression^30^ were in remission, while the other half still presented significant symptoms. Furthermore, 19.2% of the patients without this diagnosis presented criteria of depression. Such results show the need for special attention on mood symptoms during the follow-up of these patients.

The loss of independence is the central aspect of dementia and becomes more evident as the disease evolves.^3,32^ In this study, the feature was demonstrated by the performance of the patients on the Katz and Lawton scales. Moreover, this was proportional to the severity of the dementia, i.e., the most severe cases were clearly associated to more dependent subjects. Only 3.9% of the elderly patients were independent regarding instrumental activities of daily living.

Other relevant aspect of these elderly patients is their nutritional status. Loss of weight is the most prevalent nutritional problem. Moreover, it occurs insidiously, is more noticed in its advanced stage, and may lead to malnutrition and a poor prognosis. Loss of weight is associated with several factors: cognitive decline, swallowing and chewing difficulties, higher basal energy consumption, medications and immobility.^33,34^ This study showed using the MNA, that 4% of the patients were malnourished, all of these with severe dementia, while 23% were at risk of malnutrition. However, there was no statistical relevance, probably due to the few malnourished subjects. Hence, such data highlight the need for an approach addressing this issue during the follow-up of elderly patients with dementia.

By dividing the sample into two groups according to the severity of dementia, the CGA became efficient at determining the worst health condition of subjects with moderate to severe dementia. Furthermore, these patients obtained poor results in several assessed criteria. Moreover, such patients with evident cognitive impairment become more fragile, dependent, demanding huge effort by their caregivers.^35^ Being aware of the alterations of these most serious cases, can allow measures for specific interventions to be planned in order to provide improvements for the patient and their caregiver.

Achieving a broad vision for the approach regarding elderly patients with dementia, we detected underestimated clinical conditions, such as vaccination status, physical and leisure activities, risk for falls, sensorial impairments, depression and nutritional status. The subsequent intervention in those aspects that interfere in their health may provide improvements in quality of life and prognosis, and may diminish the overload imposed on their relatives.^36^

This assessment may be extended in future investigations by adding innumerous other parameters not analyzed during this study, such as: sleep alterations, social support, and environmental assessment.^9,10^

Concluding, the CGA was successfully applied among elderly outpatients with cognitive disorders. In addition, the presence of the caregiver was essential in relation to the acquisition of information about patients. The aged with moderate to severe dementia had the worst results for several assessed criteria (less leisure activities, greater risk of falls, worse performance on the MMSE, POMA and activities of daily living), which probably reflects their fragility and dependence.

Data obtained were able to efficiently characterize health condition of the studied patients, including issues barely considered in traditional assessment, such as leisure and physical activities. These results help the health team, providing assistance to promote interventions aimed at the welfare of the patient. One of the important limitations of our transversal study is that it did not include interventions for the problems detected. Future studies that associate diagnosis analysis with rehabilitation, through the use of the CGA, will be able to better assess the impact and cost-effectiveness of this tool on the quality of life of the elderly with dementia.
